# Characteristics and treatment response of polypoidal choroidal vasculopathy in highly myopic eyes

**DOI:** 10.1038/s41433-022-02251-8

**Published:** 2022-10-07

**Authors:** Wei-Lun Huang, Yun Hsia, Shih-Wen Wang, Kuo-Chi Hung, Chien-Jung Huang, Muh-Shy Chen, Tzyy-Chang Ho

**Affiliations:** 1 Department of Ophthalmology, National Taiwan University Hospital, College of Medicine, National Taiwan University, Taiwan, Republic of China; 2 Department of Ophthalmology, National Taiwan University Hospital Hsinchu Branch, Taiwan, Republic of China; 3 Graduate Institute of Clinical Medicine, College of Medicine, National Taiwan University, Taiwan, Republic of China; 4 Department of Ophthalmology, Shuang Ho Hospital, Taipei Medical University, Taiwan, Republic of China; 5 Universal Eye Clinic, Taipei, Taiwan, Republic of China; 6 Department of Ophthalmology, Fu Jen University Hospital, New Taipei City, Taiwan, Republic of China; 7 Department of Ophthalmology, Cardinal Tien Hospital, Fu Jen Catholic University, Taiwan, Republic of China

**Keywords:** Retinal diseases, Refractive errors, Macular degeneration

## Abstract

**Background:**

To compare the characteristics and treatment responses of polypoidal choroidal vasculopathy (PCV) between highly myopic and non-highly myopic eyes.

**Methods:**

This retrospective cohort study included patients diagnosed with PCV at the clinic of National Taiwan University Hospital between 2013 and 2019. The diseased eyes were grouped per refractive error and axial length at diagnosis. Imaging data were used to retrieve the PCV characteristics, and electronic medical records were used to retrieve the treatment responses.

**Results:**

Among 116 eyes with PCV, 11 eyes of 10 patients were highly myopic; seven of these patients were women. All highly myopic eyes showed a thin subfoveal choroid, while three eyes had a pachychoroid phenotype with significant focal choroidal thickening. After treatment with either intravitreal anti-vascular endothelial growth factor (VEGF) injections, photodynamic therapy (PDT), or both, best-corrected visual acuity was better in the high-myopia group at 1 year. Visual acuity at presentation and the presence of feeder vessels were found to be predictors of the visual outcome.

**Conclusions:**

In this study we reported, to the best of our knowledge, the largest cohort of PCV in highly myopic eyes to date. Female predominance, lower incidence of subretinal haemorrhage, and a thin choroid with a focal pachychoroid phenotype were found to characterise PCV in highly myopic eyes. Visual acuity transiently improved after either anti-VEGF monotherapy or combination therapy with PDT.

## Introduction

Polypoidal choroidal vasculopathy (PCV) is a choroidal disease characterised by type 1 (sub-retinal pigment epithelium) neovascularization and aneurysmal dilation, with or without a branching vascular network (BVN) [1]. In 1990, Yannuzzi et al. proposed that PCV was a subtype of neovascular age-related macular degeneration [2, 3]. However, increasing evidence supporting an association of PCV with pachychoroid spectrum disorders has been reported in recent years, and various methods for classifying the subtypes have been proposed [4, 5]. The management of PCV remains challenging, with the mainstay treatment being monotherapy or a combination of photodynamic therapy (PDT) and intravitreal anti-vascular endothelial growth factor (anti-VEGF) injections [1, 6].

PCV has a predilection for individuals of Asian descent and hyperopic eyes [1, 7]. Thus, predictably, PCV in highly myopic eyes has been uncommonly reported in the literature [8, 9]. In contrast, while it is not uncommon to find choroidal neovascularization (CNV) in eyes with high myopia, Kang et al. reviewed indocyanine green angiography (ICGA) images from a group of patients with CNV and myopia and found no case of PCV [10].

Taiwan has the highest prevalence of myopia among the young adult population [11, 12]. Since the management of PCV is known to be challenging, it being compounded by high myopia is a further challenge. In this study, we evaluated the characteristics of PCV in the case of high myopia by examining patients with PCV in our highly myopic population. Herein, we compared the characteristics and treatment responses of PCV in highly myopic eyes with those in non-highly myopic eyes. Knowing the specific characteristics and responses in cases of high myopia may help in making informed choices regarding the appropriate line of treatment.

## Subjects and methods

We retrospectively reviewed the data of patients diagnosed with PCV at the Ophthalmology Clinic of National Taiwan University Hospital, a public tertiary center in northern Taiwan, between November 2013 and September 2019. PCV was diagnosed in accordance with the EVEREST study, primarily based on the presence of early subretinal ICGA hyperfluorescence (appearing within the first 5 min of ICG dye injection) and using a second diagnostic criterion of colour fundus photography or fluorescein angiography [13]. All cases were reviewed, and their diagnoses of PCV were confirmed by the National Health Insurance Administration of Taiwan for the reimbursement of therapeutics for PCV, including PDT and anti-VEGF. The patients included in this study were aged 18 years or older and had undergone comprehensive ophthalmic examinations inclusive of the refractive status as measured with an autorefractor (KR-8800, Topcon Inc., Tokyo, Japan), best-corrected visual acuity (BCVA), optical coherence tomography (OCT) (RTVue-100; Optovue Inc., Fremont, CA, USA), and ICGA (Spectralis HRA, Heidelberg Engineering Inc., Heidelberg, Germany) upon diagnosis. Axial lengths were measured with ultrasonography (PacScan 300AP, Sonomed Inc., New York, NY, USA) or optical biometry (LS 900, Haag-Streit Group, Koeniz, Germany) in highly myopic eyes and post-operative eyes lacking refractive data before their refractive surgeries.

The patients included in the study sample were divided into four groups based on the refractive error and/or axial length of the diseased eye. High myopia is defined as a refractive error of −5.0 D or lower (according to the World Health Organization definition of 2015), or an axial length of 25.5 mm or longer in eyes with previous refractive surgery (including cataract extraction surgery). This definition was used to identify patients with highly myopic eyes. The remaining, non-highly myopic eyes were further divided into moderate myopia (−3.0 D to −4.9 D), low myopia (−0.5 D to −2.9 D), and non-myopia (>−0.5 D) groups. Pseudophakic eyes were grouped according to the preoperative refractive error.

Characteristics of PCV, including the location of the lesion, pattern of polyps, and presence of BVN and feeding vessels, were recorded using ICGA images. The greatest linear dimension of the polyps was manually measured on ICGA images. The thickness of the central macula was automatically measured with the OCT system, and the subfoveal, nasal perifoveal, and sublesional choroidal thicknesses were manually measured on OCT images. Nasal perifovea, defined as the midpoint between the fovea and the nasal optic disc margin, was used as the reference point to allow choroidal thickness comparisons between eyes with subfoveal polypoidal lesions.

The treatment of PCV applied to each patient was documented. The determinant of treatment includes visual acuity, location of the polypoid lesion, and previous treatment response; however, individual practice is mainly based on physician’s judgment. Treatments of choice included PDT, intravitreal anti-VEGF injections, and focal laser photocoagulation. Treatment outcomes are presented as the BCVA at 1 year and 2 years post diagnosis and the last clinical follow-up.

Statistical analyses were performed using commercially available software (Microsoft Office Excel 2017, Redmond, WA, USA) and a freely distributed software package (R, version 3.2.3, R Foundation for Statistical Computing, Vienna, Austria). Intergroup analyses were performed using the chi-squared test for categorical variables and one-way analysis of variance for continuous variables. The analysis of differences in treatment outcomes was performed using paired Student’s t-test and multivariate regression. *P*-values lower than 0.05 were considered statistically significant.

This retrospective study was approved by the Institutional Review Board of the National Taiwan University Hospital, which waived the requirement of informed consent, and the study was conducted in accordance with the tenets of the Declaration of Helsinki.

## Results

### Characteristics of PCV in highly myopic eyes

The groupwise demographics of the study sample are summarised in Table 1. Of the 116 eyes of 104 patients diagnosed with PCV during the study period, 11 eyes of 10 patients were highly myopic; the mean age of these patients was 59.0 ± 8.8 years. Most of the patients with high myopia were female (female patients [F]:male patients [M] = 7:3, 70.0% female); conversely, most of the patients without high myopia were men (F:M = 27:67, 28.7% female). The axial lengths of the highly myopic eyes ranged from 25.50 mm to 31.41 mm (mean: 27.63 mm). No significant difference in BCVA at baseline was noted between the groups.

**Table 1:** Demographics and baseline ophthalmic data according to the refractive error grouping.

Level of myopia	High	Moderate	Low	None	*P* value
Patients (Eyes)	10 (11)	11 (13)	32 (36)	51 (56)	
Bilaterality (%)	1 (10.0)	2 (18.2)	4 (12.5)	5 (9.8)	0.864
Age	59.0 ± 8.8	55.1 ± 13.4	60.5 ± 9.0	66.3 ± 8.9	0.001*
Female sex (%)	7 (70.0)	3 (27.3)	7 (21.9)	17 (33.3)	0.050
Refractive error (dioptres)	−7.90 ± 4.47	−3.68 ± 0.55	−1.45 ± 0.60	+1.18 ± 1.17	
BCVA at presentation (logMAR)	0.73 ± 0.72	0.64 ± 0.44	0.56 ± 0.42	0.68 ± 0.56	0.649

*BCVA* best-corrected visual acuity, *logMAR* logarithm of the minimum angle of resolution.

*P < 0.05.

The number and size of polypoidal lesions did not differ significantly with regard to the refractive error of the diseased eyes. In eyes with high myopia, BVN was noted in five eyes (45.5% eyes) and feeder vessels in two eyes (18.2%), with a ratio similar to that in non-highly myopic eyes (45.7% and 17.2%, respectively). The central retinal thickness was greater in highly myopic eyes (348.7 ± 124.4 μm, *P* = 0.043). Less subretinal haemorrhage was noted upon diagnosis in the group of highly myopic eyes (1 eye, 9.1%) than in the other refractive groups (29 eyes, 27.6%; *P* = 0.017). The anatomical characteristics are summarised in Table 2.

**Table 2:** Anatomical characteristics.

Level of myopia	High	Moderate	Low	None	*P* value
Greatest dimension (μm) of polypoid lesion	242.5 ± 76.3	212.6 ± 135.9	268.1 ± 120.3	240.2 ± 106.1	0.442
Number of polypoid lesions					0.191
Single	3	5	7	12	
2–4	5	5	7	16	
5 or more	3	3	22	28	
Site of polypoid lesions					0.018*
Subfoveal	5	5	21	14	
Perifoveal	6	5	13	37	
Extra-macular	0	3	2	5	
BVN (%)	5 (45.5)	8 (61.5)	19 (52.8)	21 (37.5)	0.315
Feeder vessel (%)	2 (18.2)	2 (15.4)	10 (27.8)	8 (14.3)	0.447
Subretinal haemorrhage (%)	1 (9.1)	4 (30.8)	4 (11.1)	21 (37.5)	0.017*
Central retinal thickness (μm)	348.7 ± 124.4	266.2 ± 93.2	260.3 ± 83.6	304.3 ± 110.6	0.043*
Choroidal thickness (μm)					
Subfoveal	124.0 ± 86.5	302.8 ± 158.6	272.9 ± 116.6	287.2 ± 120.0	0.001*
Nasal perifoveal	100.6 ± 64.7	286.5 ± 162.8	229.7 ± 125.4	242.0 ± 123.4	0.003*
Sublesional^a^	144.2 ± 84.7	294.5 ± 178.2	272.9 ± 105.0	296.1 ± 118.8	0.002*
Sattler-to-Haller ratio					
Subfoveal	0.202	0.129	0.119	0.120	0.002*
Nasal perifoveal	0.224	0.148	0.145	0.170	0.008*
Sublesional^a^	0.180	0.126	0.102	0.113	0.001*
Sublesional-to-nasal perifoveal ratio^a^	1.612	1.129	1.309	1.388	0.064
Pachychoroid (%)	3 (27.3)	6 (46.2)	16 (44.4)	34 (60.7)	0.155
Subfovea >300 μm	0	6	14	28	0.011*
Focal thickening >50 μm	3	0	3	9	0.152

*BVN* branching vascular network.

*P < 0.05.

^a^Extra-macular cases were excluded.

**Fig. 1:**
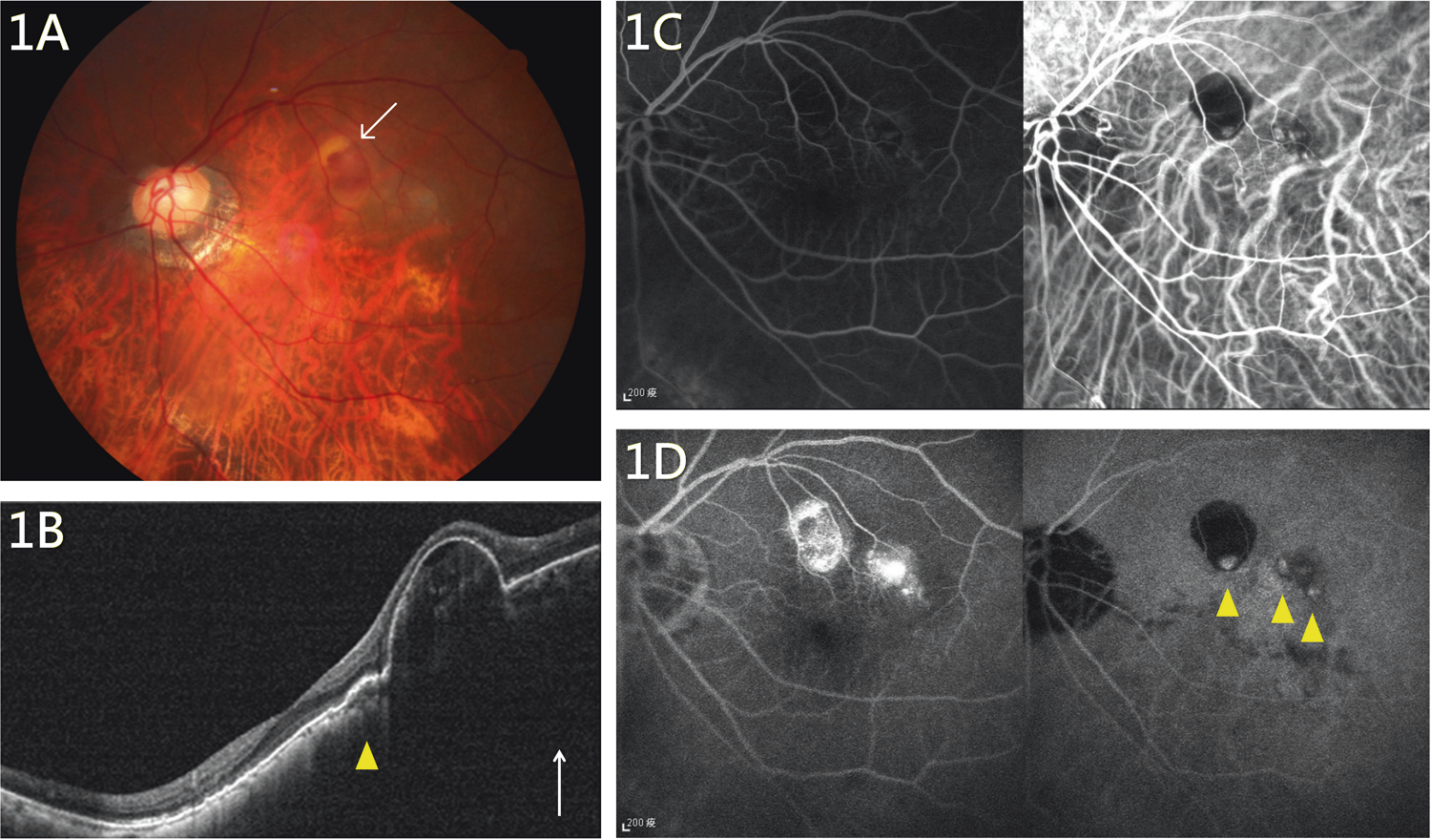
PCV in a highly myopic eye with posterior staphyloma. **A** An orange nodule (arrow) with sub-RPE haemorrhage at the superior parafovea along the posterior staphyloma border. **B** OCT showing peaked RPE detachment with parafoveal choroidal neovascularization. Relative thickening of the sublesional choroid can be observed (arrowhead). **C**, **D** ICGA reveals three polypoidal lesions at the early phase (**C**, right) and late phase (**D**, arrowheads) with leakage in the FAG (**D**, left). FAG fluorescein angiography, ICGA indocyanine green angiography, OCT optical coherence tomography, PCV polypoidal choroidal vasculopathy, RPE retinal pigment epithelium.

**Fig. 2:**
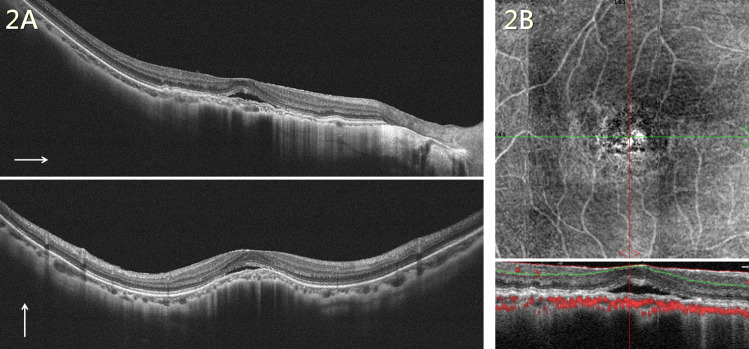
PCV in a highly myopic eye with a dome-shaped macula. **A** OCT showing choroidal neovascularization with subretinal fluid over the fovea. A dome-shaped macula can be noted in the vertical cut (lower). **B** OCT angiography showing high choroidal blood flow especially over the subfoveal area. The en face image (upper) reveals aneurysmal dilatation. OCT optical coherence tomography, PCV polypoidal choroidal vasculopathy.

Subfoveal polypoidal lesions were found in five eyes with high myopia, while the other six eyes with high myopia presented with perifoveal polypoidal lesions. Extra-macular lesions were absent in highly myopic eyes and were few in the other refractive groups. Posterior staphyloma formation was noted in five eyes (type I:II:III = 3:1:1), including one that presented with polypoidal lesions at the border of the posterior staphyloma (Fig. 1). A dome-shaped macula and concurrent subfoveal polypoidal lesions were found in one highly myopic eye with a refractive error of −15.88 D and an axial length of 31.41 mm (Fig. 2).

Highly myopic eyes with PCV showed a significantly thinner choroid and higher Sattler’s layer-to-Haller’s layer thickness ratio at all measurement points (subfovea, nasal perifovea, subpolypoidal lesion) on OCT than the non-highly myopic eyes (Table 2). The tendency of choroidal thickening over polypoidal lesions was similar in highly myopic eyes, as demonstrated by the ratio of subpolypoidal choroidal thickness and nasal parafoveal choroidal thickness (1.612, compared to 1.388 in non-highly myopic eyes, *P* = 0.064). Three highly myopic eyes (27.3%) were classified as pachychoroidal, with focal choroidal thickening greater than 50 μm, although their subfoveal choroidal thickness remained below 300 μm (Supplemental Fig. 1). Overall, the rate of the pachychoroid spectrum was similar in all refractive groups (*P* = 0.155).

### Treatment response of PCV in highly myopic eyes

Among the 116 eyes with PCV, 100 eyes received treatment and were followed up for at least 1 year. Table 3 demonstrates the treatment outcomes in each group. Most of the treated eyes (93 eyes, 93.0%), including all highly myopic eyes, received intravitreal injections of anti-VEGF. The average numbers of injections within the 1st year (2.7 ± 1.8 injections) and during the entire follow-up period (4.5 ± 3.6 injections) were similar between the groups. In the high myopia group, five eyes (67.5%) underwent photodynamic therapy either as primary or adjunctive treatment.

**Table 3:** Treatment modalities and outcomes.

Level of myopia	High *n* = 8	Moderate *n* = 10	Low *n* = 32	None *n* = 50	*P* value
IVI anti-VEGF (%)	8 (100.0)	10 (100.0)	28 (87.5)	47 (94.0)	0.577
Injections (1 year)	2.9 ± 2.1	3.3 ± 2.2	2.6 ± 1.8	2.6 ± 1.6	0.716
Injections (total)	4.4 ± 2.9	4.3 ± 3.8	5.2 ± 3.8	4.1 ± 3.5	0.641
Photodynamic therapy (%)	5 (62.5)	6 (60.0)	24 (75.0)	44 (88.0)	0.068
Courses (1 year)	1.4 ± 0.9	1.2 ± 0.8	1.0 ± 0.6	1.1 ± 0.4	0.597
Courses (total)	1.8 ± 0.8	2.0 ± 1.3	1.6 ± 0.8	1.4 ± 0.7	0.229
Focal laser therapy (%)	0 (0.0)	0 (0.0)	1 (2.9)	3 (5.7)	1.000

BCVA (logMAR)	High	Moderate	Low	None	*P* value
At presentation	0.59 ± 0.60	0.76 ± 0.39	0.61 ± 0.41	0.73 ± 0.57	0.647
At the 1-year follow-up	0.33 ± 0.28	0.59 ± 0.54	0.55 ± 0.51	0.64 ± 0.59	0.506
Change from baseline	−0.25 ± 0.37	−0.16 ± 0.46	−0.051 ± 0.43	−0.088 ± 0.49	0.687
* P* value	0.093	0.290	0.504	0.207	
At the last follow-up	0.47 ± 0.38	0.81 ± 0.84	0.52 ± 0.51	0.65 ± 0.57	0.421
Change from baseline	−0.12 ± 0.44	0.057 ± 0.77	-0.088 ± 0.45	−0.080 ± 0.49	0.858
*P* value	0.461	0.821	0.277	0.252	
Improvement ≥2 lines (%)	3 (37.5)	3 (30.0)	13 (40.6)	20 (40.0)	0.975
Worsening ≥2 lines (%)	1 (12.5)	2 (20.0)	6 (18.8)	12 (24.0)	

*BCVA* best-corrected visual acuity, *IVI* intravitreal injection, *logMAR* logarithm of the minimum angle of resolution, *VEGF* vascular endothelial growth factor.

At 1 year after treatment, the BCVA in the high myopia group was significantly better than that in the non-high myopia group (logMAR: 0.33 ± 0.28 vs. 0.69 ± 0.50, *P* = 0.034). Highly myopic eyes were found to have gained approximately two lines of visual acuity at the 1-year follow-up (logMAR: 0.59 ± 0.60 vs. 0.33 ± 0.28, *P* = 0.093), but functional improvement had declined by one line at the end of the follow-up (BCVA at the last follow-up: 0.47 ± 0.38 logMAR). No significant difference was noted between the groups at the end of the follow-up. Overall, 39 eyes (39%) had at least a two-line improvement in BCVA, whereas 21 eyes (21%) had two-line deterioration. The proportions of good and poor responders did not significantly differ between the groups.

Univariate and multivariate linear regression analyses of the 100 treated eyes were performed to identify predictors of the treatment outcome (Supplemental Tables 1 and 2). A multivariate model for BCVA changes showed that the pre-treatment BCVA was the strongest predictor (Coefficient (Coeff.) = −0.330, *P* < 0.001). While subretinal haemorrhage indicated worse initial visual acuity (Coeff. = 0.357, *P* = 0.002), it did not affect the treatment response. The presence of feeding vessels on ICGA did not predict the initial BCVA; however, it was a predictor of worse outcome at both the 1-year follow-up (Coeff. = 0.241, *P* = 0.035) and final follow-up (Coeff. = 0.252, *P* = 0.048). A higher central retinal thickness at diagnosis had a transient benefit on visual improvement at 1 year (Coeff. = −0.0009, *P* = 0.023), but this effect was not observed at the end of the follow-up. The refractive error of the eyes was not a significant outcome predictor in the multivariate model.

## Discussion

PCV has been considered a disease that affects hyperopes more than myopes [7]. Currently, however, when there is a steep increase in myopia, it is not uncommon to encounter PCV in a patient with high myopia [14]. In this study, we reported 11 highly myopic eyes with PCV, which is, to the best of our knowledge, the largest cohort to date, thus enabling for the first time, comparison of PCV characteristics between highly and non-highly myopic eyes.

Reviewing previous reports of PCV in highly myopic eyes, we found a total of eight patients reported worldwide (with 11 eyes involved), among whom, six patients (75.0%) were female (Supplemental Table 3) [8, 9, 15]. Female predominance (70.0%) in highly myopic eyes was also noted in this study, as opposed to male predominance (74.3%) in non-highly myopic eyes. Further investigation of sex differences and genetic interactions between the pathogeneses of PCV and high myopia may be needed.

Highly myopic eyes harbor several distinct anatomical features, any of which may become a weak point at the chorioretinal interface that allows PCV to occur. Mauget-Faysse et al. reported six patients (eight eyes) with PCV along with tilted disc syndrome or high myopia with staphyloma; they found that all polypoidal lesions were located at the border of the hypoplastic choroid and normal choroid [9]. In non-highly myopic eyes with inferior staphyloma and tilted disc syndrome, polypoidal lesions of PCV were also identified on the staphyloma border [16]. In the present study, however, only one of five eyes with posterior staphyloma had polypoidal lesions located at the staphyloma border. Other structural anomalies in highly myopic eyes may also contribute to the formation of PCV, as we reported in one eye with a dome-shaped macula and subfoveal polypoid lesion. The discovery of these specific locations in highly myopic eyes may add to the understanding of PCV pathogenesis in variable choroidal environments.

The discovery of the link between the pachychoroid spectrum and PCV opened a new era in PCV research [17, 18]. With an elongated axial length and thinned choroid [19], highly myopic eyes were intuitively considered to be protected from pachychoroid spectrum disease. However, Lee et al. compared the choroidal morphology in 320 PCV eyes with different subfoveal choroidal thicknesses (using 200 *μ*m as a cutoff point) and discovered that pathological dilation of the outer choroid vessels was still present in the thin choroid group [18]. Our results showed a similar trend of sublesional choroidal thickening in all groups with different refractive errors (hence, in cases with different subfoveal choroid thicknesses). Moreover, three highly myopic eyes (27.3%) were classified as pachychoroidal based on the definition of focal choroidal thickening greater than 50 *μ*m. We can conclude that the connection between the pachychoroid spectrum and PCV cannot be discarded based on the thin choroid nature of high myopia.

Functional outcomes of PCV in highly myopic eyes were found to be similar to those in non-highly myopic eyes in our study, except that a slightly better visual acuity in the high myopia group was noted at the 1-year follow-up. Two factors may explain the transient superiority in the high myopia group. First, subretinal haemorrhage, an indicator of a poor visual outcome if it occurs at the central macula, was recorded in only one (12.5%) highly myopic eye. Second, in our cohort, central retinal thickness was greater in highly myopic eyes than in non-highly myopic eyes. We propose that the retina is more susceptible to blood-retinal barrier disruption in highly myopic eyes. After therapeutic intervention, reduction in macular oedema or subretinal fluid may be more prominent, hence leading to better visual improvement in the 1st year.

This study has several limitations because of its retrospective nature and the rarity of PCV in highly myopic eyes. Many of the cases were either diagnosed before OCT angiography equipment was installed in our hospital or lacked a pre-treatment OCT angiogram; thus, analysis of the vascular pattern on OCT could not be performed. The overall treatment outcome at 1 year was inferior to that reported by a previous real-world study, which could be explained by a significantly lower number of anti-VEGF injections (2.7 ± 1.8 injections in our study vs. 5.27 ± 2.61 injections reported by Fenner et al.) [20]. The treatment protocol also greatly evolved during the study period because of several changes in the reimbursement scheme of the National Health Insurance (NHI) in Taiwan. In 2020, the expert consensus in Taiwan suggested either combined therapy of anti-VEGF and PDT as first-line therapy or anti-VEGF monotherapy with PDT added later for poor responders [21]. With the increasing accessibility of anti-VEGF by increasing its coverage by the NHI, we expect a more homogenous treatment protocol and improved treatment outcomes in the near future.

In conclusion, PCV is uncommon but not absent in highly myopic eyes. Female predominance, less subretinal haemorrhage, and a thin choroid with a focal pachychoroid phenotype were found to characterise highly myopic PCV cases. With either anti-VEGF monotherapy or combination therapy with PDT, highly myopic eyes showed an improved visual outcome at the 1-year follow-up, which carries implications for treatment recommendations.

## Summary

### What was known before

- Polypoidal choroidal vasculopathy (PCV) has a predilection for hyperopic eyes and an association with pachychoroid spectrum disorders. PCV in highly myopic eyes is uncommonly reported.

### What this study adds

- PCV in highly myopic eyes has distinct characteristics— female predominance, less subretinal haemorrhage, and thin choroid with focal pachychoroid phenotype.
- Treatment of PCV with anti-vascular endothelial growth factor injections alone or combined with photodynamic therapy is equally beneficial in highly myopic and non-highly myopic eyes.

## Supplementary information

Supplemental Figure 1

Supplemental Table 1

Supplemental Table 2

Supplemental Table 3

## Data Availability

The dataset analysed during the study is available from the corresponding author on reasonable request.
