# Supplementary material for: Characteristics and treatment response of polypoidal choroidal vasculopathy in highly myopic eyes
Source: Eye (Lond). 2022 Oct 7;37(9):1910–5. doi: 10.1038/s41433-022-02251-8 (PMC10276041; doi:10.1038/s41433-022-02251-8)
Supplement: Supplementary file 1 — Supplemental Figure 1 [file 41433_2022_2251_MOESM1_ESM.pdf]

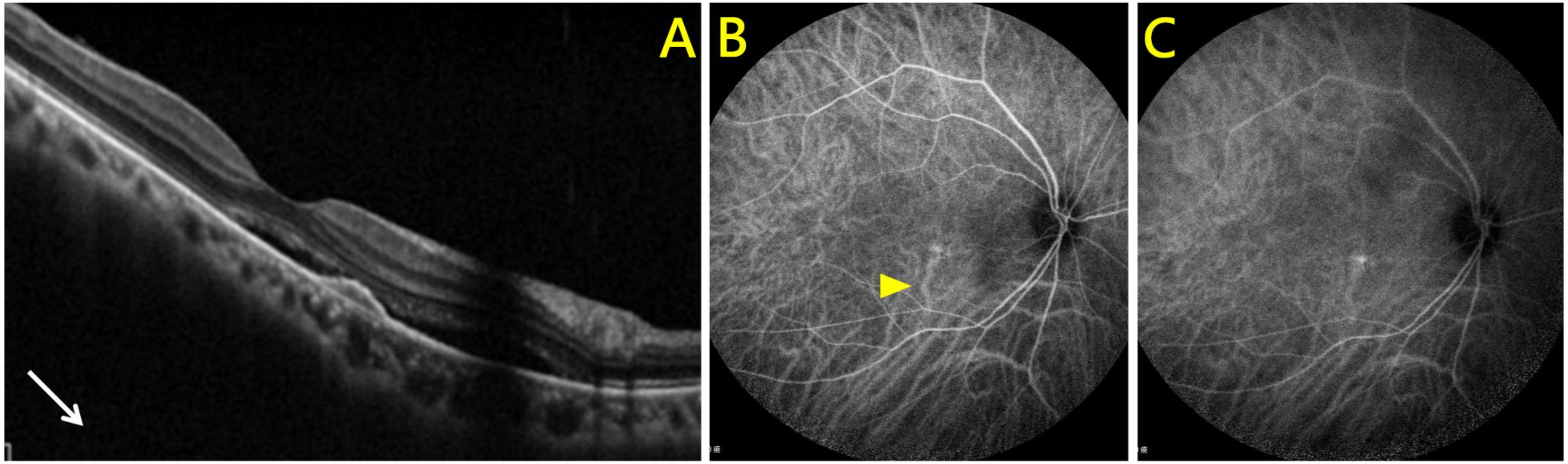

Supplemental Figure 1. PCV in a highly myopic eye with focal choroidal thickening and feeder vessel. **A:** OCT showing sub-RPE neovascularization with surrounding subretinal fluid. Signs of the pachychoroid phenotype, including marked thickening of the choroid and enlarged vessels at Haller's layer, are also presented beneath the lesion. **B:** ICGA obtained 3 minutes after injection reveals one polypoidal lesion with a feeder vessel *(arrowhead)*. **C:** ICGA at the late phase better shows the parafoveal lesion.
