# Supplementary material for: Characteristics and treatment response of polypoidal choroidal vasculopathy in highly myopic eyes
Source: Eye (Lond). 2022 Oct 7;37(9):1910–5. doi: 10.1038/s41433-022-02251-8 (PMC10276041; doi:10.1038/s41433-022-02251-8)
Supplement: Supplementary file 2 — Supplemental Table 1 [file 41433_2022_2251_MOESM2_ESM.pdf]

Supplemental Table 1: Univariate analysis.

	**BCVA at presentation**		**Change in BCVA at the 1-year follow up**§		**Change in BCVA at the last follow up**§	
**Variable**	Coeff.	*P* value	Coeff.	*P* value	Coeff.	*P* value
BCVA at presentation	-	-	-0.370	<0.0001*	-0.395	0.0001*
Age	0.011	0.023*	0.0083	0.045*	0.010	0.026*
Female sex	-0.026	0.813	-0.140	0.135	0.073	0.478
Refractive error†	-0.0058	0.766	0.0016	0.921	-0.0067	0.712
Polypoid lesion size†	0.0004	0.373	-0.0001	0.772	-0.0004	0.337
Branching vascular network†	0.148	0.145	-0.041	0.633	-0.116	0.225
Feeder vessel†	-0.166	0.186	0.241	0.022*	0.299	0.010*
Subretinal hemorrhage†	0.287	0.010*	-0.0035	0.972	0.048	0.656
Central retinal thickness‡	-0.0001	0.937	-0.0010	0.014*	-0.0008	0.070
Choroidal thickness‡						
Subfoveal	-0.0002	0.614	-0.0001	0.781	-0.0002	0.676
Nasal perifoveal	-0.0006	0.247	-0.0001	0.878	-0.0001	0.828
Sublesional	-0.0003	0.506	-0.0003	0.540	-0.0001	0.807
Pachychoroid‡	-0.116	0.297	0.028	0.763	0.012	0.908
Subfovea >300 μm	0.012	0.917	0.014	0.889	-0.068	0.531
Focal thickening >50 μm	-0.316	0.034*	0.062	0.630	0.187	0.191
IVI anti-VEGF‡	-	-	-0.013	0.590	0.026	0.045*
Photodynamic therapy‡	-	-	0.063	0.349	0.091	0.094

BCVA: best-corrected visual acuity; Coeff.: coefficient of correlation; VEGF: vascular endothelial growth factor.

*: $P < 0.05$.

†: Adjusted for age and sex.

‡: Adjusted for age, sex, and refractive error.

§: Adjusted for BCVA at presentation.
