# Supplementary material for: Characteristics and treatment response of polypoidal choroidal vasculopathy in highly myopic eyes
Source: Eye (Lond). 2022 Oct 7;37(9):1910–5. doi: 10.1038/s41433-022-02251-8 (PMC10276041; doi:10.1038/s41433-022-02251-8)
Supplement: Supplementary file 3 — Supplemental Table 2 [file 41433_2022_2251_MOESM3_ESM.pdf]

Supplemental Table 2: Multivariate analysis.

	BCVA at presentation		Change in BCVA at the 1-year follow-up		Change in BCVA at the last follow-up	
**Variable**	Coeff.	*P* value	Coeff.	*P* value	Coeff.	*P* value
BCVA at presentation	-	-	-0.330	0.0004*	-0.343	0.001*
Age	0.013	0.019*	0.0075	0.124	0.0086	0.114
Female sex	-0.066	0.552	-0.167	0.084	0.030	0.777
Refractive error	-0.021	0.309	0.0036	0.838	-0.0011	0.956
Branching vascular network	0.117	0.282	0.031	0.740	-0.020	0.851
Feeder vessel	-0.113	0.387	0.241	0.035*	0.252	0.048*
Subretinal hemorrhage	0.357	0.002*	-0.059	0.562	-0.020	0.860
Central foveal thickness	0.0001	0.916	-0.0009	0.023*	-0.0007	0.141
Pachychoroid						
Subfovea >300 *μ*m	0.025	0.828	0.056	0.565	-0.016	0.883
Focal thickening >50 *μ*m	-0.273	0.071	0.034	0.795	0.125	0.398

BCVA: best-corrected visual acuity; Coeff.: coefficient of correlation.

*: $P < 0.05$.
