# Supplementary material for: Characteristics and treatment response of polypoidal choroidal vasculopathy in highly myopic eyes
Source: Eye (Lond). 2022 Oct 7;37(9):1910–5. doi: 10.1038/s41433-022-02251-8 (PMC10276041; doi:10.1038/s41433-022-02251-8)
Supplement: Supplementary file 4 — Supplemental Table 3 [file 41433_2022_2251_MOESM4_ESM.pdf]

Supplemental Table 3: Summary of polypoidal choroidal vasculopathy in highly myopic eyes reported in the literature.

Authors [Ref]	Year	Territory	Patients (Eyes)	Female sex	Ethnicity
Kokame et al. [8]	2017	Hawaii, US	3 (4)	2	2 Chinese<br>1 Japanese
Mauget-Faysse et al. [9]	2006	France & Belgium	4 (6)	3	Caucasian
Naysan et al. [15]	2015	New York, US	1 (1)	1	Caucasian
